# Prognostic Value of Interval Between the Initiation of Neoadjuvant Treatment to Surgery for Patients With Locally Advanced Rectal Cancer Following Neoadjuvant Chemotherapy, Radiotherapy and Definitive Surgery

**DOI:** 10.3389/fonc.2020.01280

**Published:** 2020-08-21

**Authors:** Xiang-Bo Wan, Qun Zhang, Mo Chen, Yanping Liu, Jian Zheng, Ping Lan, Fang He

**Affiliations:** ^1^Department of Radiation Oncology, The Sixth Affiliated Hospital of Sun Yat-sen University, Guangzhou, China; ^2^Department of Radiation Oncology, The First Affiliated Hospital of Sun Yat-sen University, Guangzhou, China; ^3^Radiotherapy Department of Thorax and Abdomen Carcinoma, Cancer Center, The First People's Hospital of Foshan, Foshan, China; ^4^Department of Colorectal Surgery, The Sixth Affiliated Hospital of Sun Yat-sen University, Guangzhou, China

**Keywords:** locally advanced rectal cancer, neoadjuvant treatment, neoadjuvant treatment interval, prognostic value, surgery interval

## Abstract

**Background:** The addition of intensive preoperative chemotherapy and using of a longer waiting period between neoadjuvant radiotherapy and total mesorectal excision (TME) surgery lengthen the time interval from the initiation of neoadjuvant treatment to definitive surgery in patients with locally advanced rectal cancer (LARC). Here, we evaluated the prognostic value of different time intervals between the initiation of neoadjuvant treatment to TME surgery for LARC.

**Methods:** A total of 2,267 patients with LARC, who received neoadjuvant radiochemotherapy and TME surgery, between January 2010 through December 2018 were recruited. The entire cohort was divided into 4 subgroups based on total-time-to surgery, defined as the time interval between initiation of neoadjuvant treatment and TME surgery (TTS): <13 weeks (TTS-1), 13 to <15 weeks (TTS-2), 15 to <17 weeks (TTS-3), ≥17 weeks (TTS-4). Overall survival (OS), disease-free survival (DFS), distant metastasis-free survival (DMFS), and local recurrence-free survival (LRFS) rates in different TTS subgroup patients were compared, and hazard ratios (HR) for different demographic and clinicopathological variables, including TTS, were calculated to determine their prognostic significance.

**Results:** The median follow-up time was 42.0 (range, 5–162) months. The 3-year OS, DFS, DMFS, and LRFS rates were 87.0, 79.4, 80.9, and 93.8%, respectively. The varied OS, DFS, and DFMS rates were detected among these different TTS subgroups (*P* = 0.010, *P* < 0.001, and *P* < 0.001, respectively). Particularly, the lower survival outcome was mainly observed at patients in the shortest TTS group (TTS-1). Cox regression analysis confirmed that the only significant positive independent prognostic factor for 3-year DFS was a longer TTS (TTS 2–4 vs. TTS-1; HR 0.884, 95% CI 0.778–0.921, *P* < 0.001), while the significant negative independent prognosticfactors were moderate to poor tumor differentiation (vs. well-differentiated; HR 1.191, 95% CI 1.004–1.414, *P* = 0.045) and clinical N1-2 stage (vs. N0 stage; HR 1.190, 95% CI 1.052–1.347, *P* = 0.006).

**Conclusion:** For patients with LARC, an interval between the initiation of neoadjuvant treatment and TME surgery of longer than 13 weeks is associated with favorable disease-free survival.

## Introduction

In the past few decades, the combination of neoadjuvant radiochemotherapy and total mesorectal excision (TME) surgery has markedly reduced the local recurrence rate, and serving as the standard therapeutic regimen for patients with locally advanced rectal cancer (LARC) (1, 2). However, the predominant cause of disease relapse in patients receiving this treatment is distant metastases, with the 5-year distant relapse rate estimated to be about 35% (3–5).

To attempt to address this issue, patients with LARC have been given additional adjuvant chemotherapy (ACT) after neoadjuvant radiochemotherapy and TME surgery. However, accumulated evidences showed that the effectiveness of ACT in LARC patients was limited (6–8). Considering that many patients declined or failed to complete ACT, efforts have been made to identify other more intensive systemic treatment approaches that can be completely administered in the neoadjuvant setting, with the aims of both downstaging locally advanced tumor and reducing the risks of metastatic disease prior to surgery. One selection was the administration of induction chemotherapy, followed by radiotherapy, and then subsequent consolidation chemotherapy, all prior to TME surgery. Another approach involved the use of total neoadjuvant therapy (TNT), in which all planned radiotherapy and intensive chemotherapy was delivered in the preoperative setting. Compared to ACT, these modified treatment strategies had a superior patient compliance rate, the long-term survival outcome results was however not yet to be published (9–12). Significantly, using of more intensive neoadjuvant chemotherapy (NCT) resulted in longer intervals between the beginning of neoadjuvant treatment and TME surgery. However, the appropriate period between the initiation of neoadjuvant therapy and TME surgery in patients with LARC remains unclear.

Likewise, there is debate about the optimal interval between the completion of neoadjuvant radiotherapy and TME surgery in patients with LARC. To achieve a high pathologic complete response (pCR) rate, which is considered a surrogate marker of favorable oncological outcomes (13), there has been a trend toward prolonging the interval between radiotherapy and TME surgery, especially when part of the aim is organ preservation. For the underlying reason, the amount of tumor regression after radiotherapy has been shown to be time-dependent (14). Taken Lyon R90-01 trial for example, a longer interval (6–8 weeks vs. up to 2 weeks) between radiotherapy and surgery was correlated with increased pCR rates, though there was no difference in long-term overall survival (OS) between two subgroups (15). Although the guideline of the National Comprehensive Cancer Network (NCCN) recommends that TME surgery should be done between 5 and 12 weeks after chemoradiotherapy (16), the GRECCAR-6 trial indicated that a longer interval of 11 weeks (vs. 7 weeks) between chemoradiotherapy and surgery resulted in increased postoperative morbidity and a poorer quality of TME surgery, without an increased pCR rate (17). Additionally, data collected from the United States National Cancer Database demonstrated that a delay in surgery of more than 8 weeks after neoadjuvant radiotherapy was associated with a higher rate of positive surgical margins as well as a decreased rate of survival in patients with LARC (18, 19). Given these somewhat contradictory results, the optimal period between neoadjuvant radiotherapy and TME surgery in LARC patients remains unclear.

In this study, we included a large size of LARC patients, who treated with intensive chemotherapy, radiotherapy, and TME surgery, with the aim of determining the impact of different time intervals from the initiation of neoadjuvant treatment to surgery, and of different waiting periods from the end of radiotherapy to surgery, on survival outcome and pCR rates.

## Methods

Between January 2010 and December 2018, patients with newly diagnosed, biopsy-proven, non-metastatic LARC treated were included in this study. All patients were staged according to the 7th edition of the American Joint Commission on Cancer (AJCC) staging system (20). Patients with locally advanced rectal cancers were defined as stage II (T3-4N0) or stage III (T1-4N1-2) by magnetic resonance imaging (MRI), computed tomography (CT), and/or endorectal ultrasonography (EUS). All patients had adenocarcinomas with a distal border located <12 cm from the anal verge. Patients were excluded who had a previous history of any type of cancer, treatment duration from the beginning of neoadjuvant treatment to TME surgery of more than 9 months or waiting period from the end of radiotherapy to TME surgery of <4 weeks. The Institutional Review Board of the Sixth Affiliated Hospital at Sun Yat-sen University approved this retrospective study.

### Neoadjuvant and Adjuvant Treatment

All patients were treated with neoadjuvant intensity-modulated radiotherapy (IMRT). For each patient, the gross tumor volume (GTV) received 50 Gy in 25 fractions at 2.0 Gy per fraction, while the clinical target volume (CTV) received 45 Gy in 25 fractions at 1.8 Gy per fraction.

In addition to radiotherapy, all patients received neoadjuvant induction (before radiotherapy), concurrent (during radiotherapy), and/or consolidation (after radiotherapy) chemotherapy, based on the treatment guidelines for locally advanced rectal cancer. Neoadjuvant chemotherapy (NCT) consisted of a fluoropyrimidine-based regimen, determined at the discretion of the multi-disciplinary cancer team, involving one of the following: folinic acid, fluorouracil, and oxaliplatin (FOLFOX); capecitabine and oxaliplatin (CAPOX); capecitabine (Xeloda); or folinic acid, fluorouracil, oxaliplatin, and rinotecan (FOLFOXIRI).

A majority of the patients in this cohort also received a fluoropyrimidine-based ACT regimen, which was determined at the discretion of the multi-disciplinary cancer team. The same team also made the decision of the specific number of cycles of both NCT and ACT given to each patient. Total neoadjuvant therapy (TNT) in this study was defined as receiving 8 or more cycles of NCT and no ACT.

### Total-Time-to-Surgery (TTS) and Waiting-Period-After-Radiotherapy (WPR)

Total-time-to-surgery (TTS) was defined as the time from the initiation of any neoadjuvant treatment to the date of TME surgery. The median TTS was 101 days (range, 58–265 days), and the interquartile TTS cut points were at 89, 101, and 117 days, which we approximated as 13, 15, and 17 weeks, respectively. Using these fixed cut points, patients were then divided into 4 groups: those who had TME surgery <13 weeks from the initiation of treatment (TTS-1), those who had TME surgery from 13 to <15 weeks from the initiation of treatment (TTS-2), those who had TME surgery from 15 to <17 weeks from the initiation of treatment (TTS-3), and those who had surgery 17 or more weeks from the initiation of treatment (TTS-4).

Waiting-period-after-radiotherapy (WPR) was defined as the time from the end of radiotherapy to the date of TME surgery. The median WPR was 56 days (range, 28–207 days). Patients were arbitrarily divided into 5 groups for every 2 weeks of WPR: those with waiting periods of 4 to <6 weeks (WPR-1), those with waiting periods of 6 to <8 weeks (WPR-2), those with waiting periods of 8 to <10 weeks (WPR-3), those with waiting periods of 10 to <12 weeks (WPR-4), and those with waiting period of 12 or more weeks (WPR-5).

### Follow-Up

Follow-up duration was defined as the time from the first day of treatment to either the date of last examination or the date of death. Patients were routinely assessed at 3-month intervals during the first 3 years and at 6-month intervals thereafter or until death. Primary endpoints used in this study included the following: overall survival (OS), measured as the time from the initiation of treatment to death from any cause; disease-free survival (DFS), measured as the time from TME surgery to the first disease relapse at any site; distant metastasis-free survival (DMFS), measured as the time from TME surgery to the first distant relapse (recurrence outside the pelvis); and locoregional relapse-free survival (LRFS), measured as the time from TME surgery to the first locoregional relapse (recurrence within the pelvis).

Other clinicopathological characteristics evaluated in this study included the following: pathologic complete response (pCR), defined as the absence of viable adenocarcinoma cells in the TME surgical specimen (ypT0N0); pathologic stage after neoadjuvant therapy and TME surgery (ypTN stage); downstaging, defined as stage ypT0-2N0 after TME surgery; and surgical specimen pathology results (vascular invasion, neural invasion, and surgical margin status and measurement).

### Statistical Methods

The χ^2^-test was used to compare the distributions of assorted demographic and clinicopathological characteristics in different TTS subgroups. Kaplan-Meier survival curves were used to compare patient outcomes (OS, DFS, DMFS, and LRFS) among different TTS and WPR subgroups. Statistical differences between curves were calculated using the log-rank test. The multivariate Cox proportional hazards model was utilized to estimate the hazard ratios (HR) and 95% confidence intervals (CI) for different demographic and clinicopathological characteristics, so that HRs for OS, DFS, and DMFS equated to the relative risks of death, disease relapse, and distant metastasis, respectively. All *P-*values were two-sided, and a *P* < 0.05 was considered statistically significant. Statistical analyses were performed with the Statistical Package for the Social Sciences (SPSS, version 24.0; SPSS, Inc, Chicago, IL).

## Results

Totally, 2,267 patients with LARC were included in this study. Of whom, 1,540 (67.9%) were men and 727 (32.1%) were women, and the median age was 56.0 years (range, 15–87 years) (Table 1). Particularly, only 34 patients received TNT (3 in the TTS-3 group, 31 in the TTS-4 group). The clinicopathological factors of gender, as well as tumor differentiation and distance from the anal verge, and patients receiving TNT did not differ significantly among 4 TTS subgroups. Conversely, the age distributions of patients in the TTS groups was differed significantly, such that those with longer TTS (TTS-3 and TTS-4) were more frequently younger (56 years and younger) than older (57 years and older), whereas those in the shorter TTS-1 and TTS-2 subgroups were more frequently older than younger (*P* = 0.03). Additionally, cT stage, cN stage, and cTNM stage distributions of patients in the TTS groups were also differed significantly, that those with longer TTS (TTS-3 and TTS-4) were more frequently to have advanced cT stage (cT4 vs. cT1-3, *P* < 0.001), cN stage (cN+ vs. cN0, *P* = 0.005), and cTNM stage (III vs. II, *P* < 0.001).

**Table 1:** Demographic and clinicopathological characteristics, by total-time-to-surgery (TTS)^a^, of 2,267 patients with locally advanced rectal cancer, January 2010 through December 2018.

**Characteristics**		**Total-time-to-surgery (TTS)**^****a****^				
	**Total patients**	**TTS-1**	**TTS-2**	**TTS-3**	**TTS-4**	***P***
		**<13 weeks**	**13–15 weeks**	**15–17 weeks**	**≥ 17 weeks**	
		**(*n =* 657)**	**(*n =* 616)**	**(*n =* 465)**	**(*n =* 529)**	
		**No. (%)**	**No. (%)**	**No. (%)**	**No. (%)**	
Age, years (median, 56)						0.030
≤ 56	1,143	317 (48.2)	297 (48.2)	246 (52.9)	283 (53.5)	
>56	1,124	340 (51.8)	319 (51.8)	219 (47.1)	246 (46.5)	
Gender						0.106
Male	1,540	433 (65.9)	416 (67.5)	320 (68.8)	371 (70.1)	
Female	727	224 (34.1)	200 (32.5)	145 (31.2)	158 (29.9)	
Clinical T stage						<0.001
cT1	6	0 (0.0)	2 (0.3)	2 (0.4)	2 (0.4)	
cT2	83	22 (3.3)	27 (4.4)	15 (3.2)	19 (3.6)	
cT3	1,427	445 (67.7)	407 (66.1)	287 (61.7)	288 (54.4)	
cT4	751	190 (28.9)	180 (29.2)	161 (34.6)	220 (41.6)	
Clinical N stage						0.005
cN0	447	159 (24.2)	121 (19.6)	82 (17.6)	85 (16.1)	
cN1	973	259 (39.4)	265 (43.0)	224 (48.2)	225 (42.5)	
cN2	847	239 (36.4)	230 (37.3)	159 (34.2)	219 (41.4)	
Clinical TNM stage						<0.001
II	447	159 (24.2)	121 (19.6)	82 (17.6)	85 (16.1)	
III	1,820	489 (75.8)	495 (80.4)	383 (82.4)	444 (83.9)	
Distal tumor distance from anal verge, cm						0.368
0–5	1,245	381 (58.0)	325 (52.8)	251 (54.0)	288 (54.4)	
>5– ≤ 10	899	241 (36.7)	255 (41.4)	192 (41.3)	211 (39.9)	
>10	66	18 (2.7)	23 (3.7)	10 (2.2)	15 (2.8)	
Unknown/missing	57	17 (2.6)	13 (2.1)	12 (2.6)	15 (2.8)	
Tumor differentiation						0.177
Highly-differentiated	287	74 (11.3)	95 (15.4)	54 (11.6)	64 (12.1)	
Moderately-differentiated	1,612	482 (73.4)	434 (70.5)	330 (71.0)	366 (69.2)	
Poorly-differentiated	368	101 (15.4)	87 (14.1)	81 (17.4)	99 (18.7)	
Neoadjuvant chemotherapy (NCT) cycles						<0.001
0–3	1,365	609 (92.7)	419 (68.0)	184 (39.6)	153 (28.9)	
≥4	902	48 (7.3)	197 (32.0)	281 (60.4)	376 (71.1)	
Total neoadjuvant therapy (TNT)^b^	34	0 (0.0)	0 (0.0)	3 (8.8)	31 (91.2)	0.154
Adjuvant chemotherapy (ACT) cycles						<0.001
0	461	120 (18.3)	101 (16.4)	95 (20.4)	145 (27.4)	
1–4	993	230 (35.0)	241 (39.1)	235 (50.5)	287 (54.3)	
≥5	813	307 (46.7)	274 (44.5)	135 (29.0)	97 (18.3)	

a Total-time-to-surgery (TTS) defined as time from initiation of neoadjuvant treatment to date of surgery.

b *Total neoadjuvant therapy (TNT) defined as receiving more than 8 cycles of neoadjuvant chemotherapy (NCT) and no adjuvant chemotherapy (ACT)*.

Significantly, the distribution of patients among these 4 TTS subgroups, according to whether they received above or below the median number of NCT and ACT cycles, were differed greatly, shown as that those with longer TTS (TTS-3 and TTS-4) was prone to receive more NCT (4 or more cycles vs. 0–3 cycles, *P* < 0.001) and less ACT (0–4 cycles vs. 5 or more cycles, *P* < 0.001) (Table 1). Nevertheless, the total cycles of all chemotherapy (combining NCT and ACT) given to patients were similar across all TTS subgroups (range, 6.5 ± 2.8 to 7.3 ± 2.9, all *P* > 0.05) (Table 2).

**Table 2:** Neoadjuvant, adjuvant, and total chemotherapy cycles, by total-time-to-surgery (TTS)^a^, in 2,267 patients with locally advanced rectal cancer, January 2010 through December 2018.

**TTS^**a**^ group**	**Total patients**	**Neoadjuvant chemotherapy**		**Adjuvant chemotherapy**		**Total chemotherapy**	
		**No. of patients (%)**	**No. of cycles mean, SD**	**No. of patients (%)**	**No. of cycles mean, SD**	**No. of patients (%)**	**No. of cycles mean, SD**
TTS-1 (<13 weeks)	657	653 (99.4)	2.5 ± 0.8	537 (81.7)	4.0 ± 2.6	654 (99.5)	6.5 ± 2.8
TTS-2 (13 to <15 weeks)	616	613 (99.5)	3.2 ± 1.3	515 (83.6)	4.0 ± 2.5	615 (99.8)	7.1 ± 3.1
TTS-3 (15 to <17 weeks)	465	454 (97.6)	3.7 ± 1.5	370 (79.6)	3.2 ± 2.4	465 (100.0)	7.0 ± 2.9
TTS-4 (≥17 weeks)	529	528 (99.8)	4.6 ± 2.2	384 (72.6)	2.7 ± 2.2	528 (99.8)	7.3 ± 2.9

a *Total-time-to-surgery (TTS) defined as time from initiation of neoadjuvant treatment to date of surgery*.

Of these 2,267 patients, 555 (24.5%) cases achieved a pCR (Table 3). Patients in the subgroups with longer TTS had significantly higher pCR rates: 18.3% in TTS-1, 24.2% in TTS-2, 26.0% in TTS-3, and 31.2% in TTS-4 (*P* < 0.001). Similarly, patients in the subgroups with progressively longer TTS also had significantly higher rates of downstaging (to ypT0-2N0): 23.1% in TTS-1, 29.2% in TTS-2, 31.4% in TTS-3, and 35.3% in TTS-4 (*P* < 0.001). The rates of positive surgical margins in all TTS subgroups were low (0.3% in TTS-1, 0.6% in TTS-2, 0% in TTS-3 and TTS-4) and did not differ significantly among the TTS subgroups (*P* = 0.121).

**Table 3:** Clinicopathological outcomes, by total-time-to-surgery (TTS)^a^, of 2,267 patients with locally advanced rectal cancer, January 2010 through December 2018.

**Outcomes**		**Total-time-to-surgery (TTS)**^****a****^				
	**Total patients**	**TTS-1**	**TTS-2**	**TTS-3**	**TTS- 4**	***P***
		**<13 weeks**	**13–15 weeks**	**15–17 weeks**	**≥ 17 weeks**	
		**(*n =* 657)**	**(*n =* 616)**	**(*n =* 465)**	**(*n =* 529)**	
		**No. (%)**	**No. (%)**	**No. (%)**	**No. (%)**	
Pathologic complete response (pCR)						<0.001
Yes	555	120 (18.3)	149 (24.2)	121 (26.0)	165 (31.2)	
No	1,712	537 (81.7)	467 (75.8)	344 (74.0)	364 (68.8)	
ypT stage^b^						<0.001
ypT0	580	128 (19.5)	156 (25.3)	124 (26.7)	172 (32.5)	
ypT1	130	39 (5.9)	37 (6.0)	28 (6.0)	26 (4.9)	
ypT2	504	143 (21.8)	138 (22.4)	111 (23.9)	112 (21.2)	
ypT3	783	270 (41.1)	217 (35.2)	137 (29.5)	159 (30.1)	
ypT4	270	77 (11.7)	68 (11.0)	65 (14.0)	60 (11.3)	
ypN stage^b^						0.001
ypN0	1,761	484 (73.7)	468 (76.0)	377 (81.1)	432 (81.7)	
ypN1	407	138 (21.0)	124 (20.1)	67 (14.4)	78 (14.7)	
ypN2	99	35 (5.3)	24 (3.9)	21 (4.5)	19 (3.6)	
ypTN stage^b^						<0.001
ypT0N0	555	120 (18.3)	149 (24.2)	121 (26.0)	165 (31.2)	
I	110	32 (4.9)	31 (5.0)	25 (5.4)	22 (4.2)	
II	1,098	333 (50.7)	287 (46.6)	232 (49.9)	246 (46.5)	
III	504	172 (26.2)	149 (24.2)	87 (18.7)	96 (18.1)	
Downstaging (to stage ypT0-2N0)						<0.001
Yes	665	152 (23.1)	180 (29.2)	146 (31.4)	187 (35.3)	
No	1,602	505 (76.9)	436 (70.8)	319 (68.6)	342 (64.7)	
Vascular invasion						0.304
Negative	2,211	648 (98.6)	599 (97.2)	444 (95.5)	520 (98.3)	
Positive	56	9 (1.4)	17 (2.8)	21 (4.5)	9 (1.7)	
Neural invasion						0.469
Negative	2,158	628 (95.6)	584 (94.8)	448 (96.3)	498 (94.1)	
Positive	109	29 (4.4)	32 (5.2)	17 (3.7)	31 (5.9)	
Surgical margin						0.121
Negative	2,261	655 (99.7)	612 (99.4)	465 (100.0)	529 (100.0)	
Positive	6	2 (0.3)	4 (0.6)	0 (0.0)	0 (0.0)	
Circumferential resection margin, mm						0.696
≤ 1	2,246	652 (99.2)	608 (98.7)	460 (98.9)	526 (99.4)	
>1	21	5 (0.8)	8 (1.3)	5 (1.1)	3 (0.6)	

a Total-time-to-surgery (TTS) defined as time from initiation of neoadjuvant treatment to date of surgery.

b *yp stage is pathologic stage after neoadjuvant treatment and surgical resection*.

The median follow-up duration for the entire cohort was 42.0 months (range, 5–162 months). The overall 3-year OS, DFS, DMFS, and LRFS rates for all patients were 87.0, 79.4, 80.9, and 93.8%, respectively. Univariate analysis displayed that tumor differentiation, number of NCT cycles, and TTS was the prognostic factors of OS (*P* < 0.001, *P* = 0.001, and *P* = 0.011, respectively); and clinical N stage, tumor distance from anal verge, tumor differentiation, and TTS was correlated with DFS (*P* = 0.030, *P* = 0.032, *P* = 0.001, and *P* < 0.001, respectively) and DMFS (*P* = 0.030, *P* = 0.039, *P* = 0.009, and *P* < 0.001, respectively). However, age, gender, clinical T stage, clinical TNM stage, and WPR did not correlate significantly with OS, DFS, or DMFS (Supplementary Table 1).

On initial multivariate analysis, only tumor differentiation and TTS were correlated with OS (*P* < 0.001 and *P* < 0.001, respectively), while cN stage, tumor differentiation, and TTS were correlated significantly with DFS (*P* = 0.023, *P* = 0.001, and *P* < 0.001, respectively) and DMFS (*P* = 0.021, *P* = 0.008, and *P* < 0.001, respectively) (Supplementary Table 1). Furthermore, compared to that of shorter interval TTS-1 subgroup, patients with longer interval time (TTS-2, TTS-3, and TTS-4 subgroups) had lower HR for 3-year relapse: 0.798 (95% CI 0.636–1.002, *P* = 0.052), 0.547 (95% CI 0.413–0.724, *P* < 0.001), and 0.680 (95% CI 0.525–0.880, *P* = 0.003), respectively.

**Figure 1:**
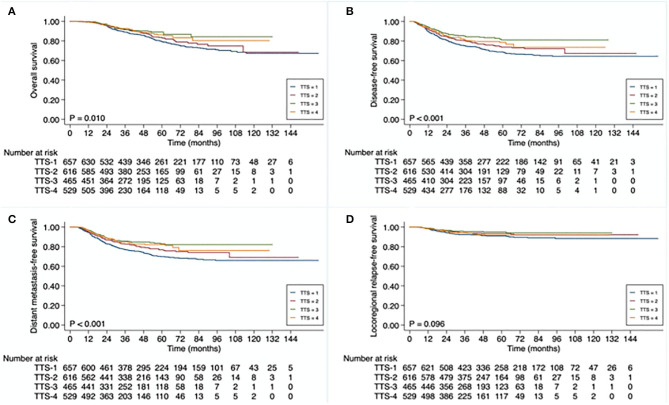
Kaplan-Meier curve analysis of **(A)** overall survival, **(B)** disease-free survival, **(C)** distant metastasis-free survival, and **(D)** locoregional relapse-free survival **(D)**, based on total-time-to-surgery (TTS), of 2,267 patients with locally advanced rectal cancer. TTS defined as time from initiation of neoadjuvant treatment to date of surgery: <13 weeks (TTS-1), 13 to <15 weeks (TTS-2), 15 to <17 weeks (TTS-3), ≥17 weeks (TTS-4).

The Kaplan-Meier survival curve analysis confirmed that OS, DFS, and DFMS rates differed significantly between different TTS subgroups (*P* = 0.010, *P* < 0.001, and *P* < 0.001, respectively). Moreover, the evident survival differences were mainly observed between TTS-1 subgroup and TTS-2/TTS-3/TTS-4 subgroups (Figure 1). As shown, the 3-year DFS rates were 74.7% for patients in the TTS-1 group, while was 79.8% for those in the TTS-2 group, 84.4% for those in the TTS-3 group, and 80.5% for those in the TTS-4 group.

**Figure 2:**
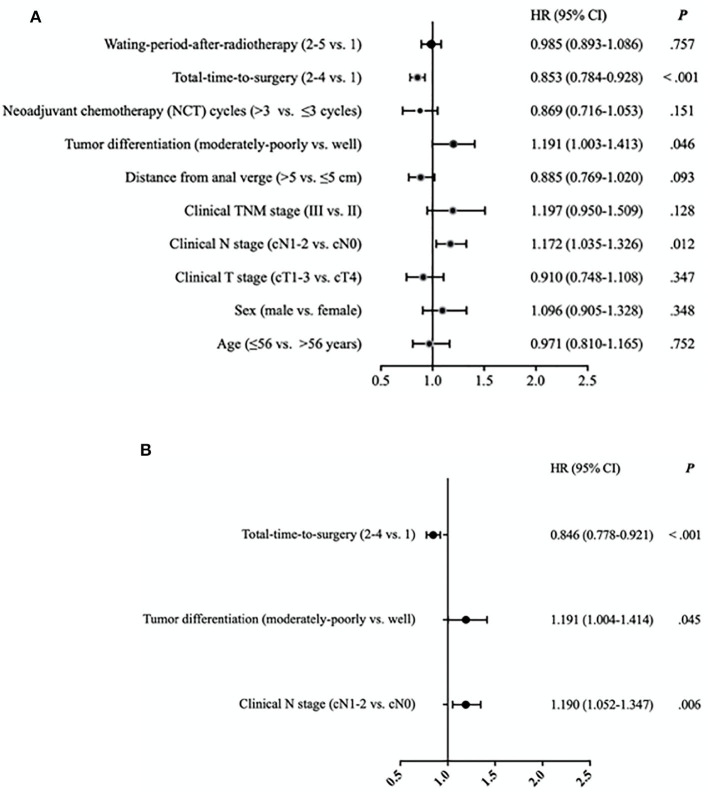
**(A)** Univariate analysis of risk of disease relapse (disease-free survival) for 2,267 patients with locally advanced rectal cancer. Hazard ratios (HR) with 95% confidence intervals (CI) on 3-year disease-free survival equate to relative risk of disease relapse. Waiting-period-after-radiotherapy (WPR) defined as time from end of radiotherapy to date of surgery: 4 to <6 weeks (WPR-1), 6 to <8 weeks (WPR-2), 8 to <10 weeks (WPR-3), 10 to <12 weeks (WPR-4), and ≥12 weeks (WPR-5). Total-time-to-surgery (TTS) defined as time from initiation of neoadjuvant treatment to date of surgery: <13 weeks (TTS-1), 13 to <15 weeks (TTS-2), 15 to <17 weeks (TTS-3), and ≥17 weeks (TTS-4). **(B)** Multivariate analysis of risk of disease relapse (disease-free survival) for 2,267 patients with locally advanced rectal cancer. Hazard ratios (HR) with 95% confidence intervals (CI) on 3-year disease-free survival equate to relative risk of disease relapse. Total-time-to-surgery (TTS) defined as time from initiation of neoadjuvant treatment to date of surgery: <13 weeks (TTS-1), 13 to <15 weeks (TTS-2), 15 to <17 weeks (TTS-3), and ≥17 weeks (TTS-4).

On the second univariate (Figure 2A) and multivariate analysis (Figure 2B), using some different referents and focusing only on 3-year DFS, the only significant clinically positive independent prognostic factor for 3-year DFS that remained was longer TTS (TTS 2–4 vs. TTS-1; HR 0.884, 95% CI 0.0.778–0.921, *P* < 0.001). The significant clinically negative independent prognostic factors were moderate to poor tumor differentiation (vs. well-differentiated; HR 1.191, 95% CI 1.004–1.414, *P* = 0.045) and cN1-2 stage (vs. cN0; HR 1.190, 95% CI 1.052–1.347, *P* = 0.006).

**Figure 3:**
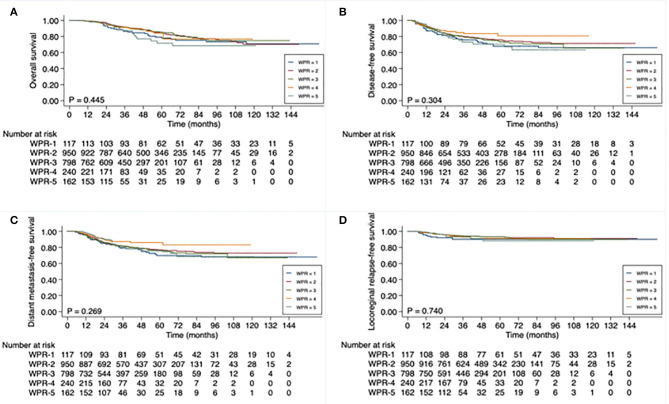
Kaplan-Meier curve analysis of **(A)** overall survival, **(B)** disease-free survival, **(C)** distant metastasis-free survival, and **(D)** locoregional relapse-free survival **(D)**, based on waiting-period-after-radiotherapy (WPR), of 2,267 patients with locally advanced rectal cancer. WPR defined as time from end of neoadjuvant radiotherapy to date of surgery: 4 to <6 weeks (WPR-1), 6 to <8 weeks (WPR-2), 8 to <10 weeks (WPR-3), 10 to <12 weeks (WPR-4), and ≥12 weeks (WPR-5).

As with patients in subgroups with longer TTS, patients in subgroups with progressively longer WPR also had significantly higher rates of pCR: 19.7% in WPR-1, 23.7% in WPR-2, 24.1% in WPR-3, 25.8% in WPR-4, and 32.7% in WPR-5 (*P* = 0.025). However, despite the increased pCR rates was associated with longer WPR, WPR did not significantly correlate with any of the survival endpoints (Supplementary Table 1; Figure 3).

## Discussion

In this study, we reported the prognostic value of total-time-to-surgery (TTS) in patients with locally advanced rectal cancer (LARC) who have received intensive neoadjuvant and/or adjuvant chemotherapy in addition to neoadjuvant radiotherapy and TME surgery, using multiple different survival rates as clinical endpoints. We identified TTS as an independent prognostic factor for both 3-year DFS and 3-year DMFS in patients with LARC. Specifically, our results showed that patients with LARC achieved better survival outcomes when they underwent TME surgery 13 or more weeks after the initiation of neoadjuvant treatment.

In the past, the standard approach for patients with LARC was concurrent neoadjuvant chemoradiotherapy, followed by TME surgery and then ACT. However, with this strategy distant metastases remained a common reason of failure (3–5). To address this, intensified chemotherapy has been administered, either by including more cytotoxic drugs in each chemotherapy cycle, or more total cycles of chemotherapy, prior to TME. This can be done by splitting the chemotherapy between NCT and ACT, or alternatively by giving all chemotherapy as NCT, which some have named as total neoadjuvant therapy (TNT). NCT certainly has some theoretical benefits over ACT, including potentially improved compliance with chemotherapy, earlier eradication of occult metastases, prompt identification of non-responders, and increased resectability of the primary tumor.

An example of adding cytotoxic drugs was the CAO/ARO/AIO-04 multicenter study, which involved patients with LARC who received neoadjuvant chemoradiotherapy, surgery, and ACT with or without oxaliplatin, and demonstrated 3-year DFS rates of 75.9 and 71.2%, respectively (21). TNT has been evaluated in a meta-analysis which involved 28 studies and demonstrated 3-year DFS and OS rates of 67 and 78.9%, respectively, in patients receiving TNT for LARC (22). Our FORWARC study, which also used an intensified NCT regimen in the preoperative setting, showed that 3-year DFS rates for fluorouracil plus radiotherapy, mFOLFOX6 plus radiotherapy, and mFOLFOX6 alone, were 72.9, 77.2, and 73.5%, respectively (23). Our present findings are similar to these reported studies, showing a 3-year DFS of 79.3% in all patients who received intensive NCT and/or ACT, in addition to radiotherapy and TME surgery.

While beneficial, the expanding use of NCT in the preoperative setting in recent years has resulted in an increase in the time from the initiation of neoadjuvant treatment to definitive surgery (24). For example, Garcia-Aguilar et al. (25) studied the impact of adding cycles of neoadjuvant chemotherapy on the time from the start of chemoradiation to surgery in patients with LARC (25). They explored the effect of adding 0, 2, 4, and 6 cycles of consolidation chemotherapy, after neoadjuvant chemoradiotherapy and before surgery, on pCR. They found that the mean total-time-to surgery for each of these 4 groups was 14.2, 17.1, 21.0, and 25.2 weeks, respectively. They also found 5-year DFS rates for each of these groups of 50, 81, 86, and 76%, respectively. Because of their study design, it was not possible to conclude that the survival benefits resulted solely from the longer intervals (26). However, their results were in line with our findings, that the 5-year DFS rate for patients with a TTS <13 weeks was significantly inferior to the rates for patients with longer TTS. These results provide evidence that a longer TTS may play an important role in the success of intensive treatment regimens in patients with LARC.

The goals of giving NCT to patients with LARC in the preoperative setting include the eradication of occult micro-metastases and the downsizing of locoregional disease. Nevertheless, it is not possible in the majority of patients to eliminate all of the primary tumor without surgery. Indeed, we found that only 24.5% of patients in our study achieved pCR. However, the rate of pCR progressively increased with longer TTS, up to 31.2% in patients with a TTS of 17 weeks or more. Despite this, we noticed a slight decline in the 3-year DFS rates in patients in the TTS-4 subgroup (80.5%) compared to those in the TTS-3 subset (84.4%). Evidence exists that tumor cell repopulation might accelerate after chemotherapy or radiotherapy (27). Consequently, markedly prolonged time intervals between neoadjuvant treatment and definitive surgery may actually increase the risk of disease progression. Thus, there is still work to do to identify the optimal TTS, one that allows patients to receive the maximum therapeutically beneficial systemic neoadjuvant treatment while also avoiding an excessive delay that may result in disease progression.

It has been suggested that the waiting period between the completion of radiotherapy and the performance of surgery, which represents a component of TTS, may also play a role in determining survival rates in patients with LARC. Using pCR as a marker for oncological success, the Lyon R90-01 trial established a generally accepted waiting period after radiotherapy of 6–8 weeks (15), and the NCCN guideline recommended a range of 5–12 weeks as a proper interval (15, 16). However, neither of these addressed the impact of this waiting period on survival. In our study, the pCR rate increased when the WPR became longer, ranging from 19.7% up to 32.7% for a waiting period between radiotherapy and surgery of 12 weeks or longer. At the same time, we also found that WPR did not correlate with 3-year DFS. Therefore, the optimal waiting period between neoadjuvant radiotherapy and TME surgery remains to be clarified.

This study has several limitations. It included variations in patient baseline clinicopathological characteristics among the 4 different TTS subgroups. For example, the groups with longer TTS included a significantly larger proportion of patients who had advanced cTNM stages. Theoretically, this should correlate with inferior survival results in the groups with longer TTS, while the survival results were actually superior in these subgroups. Moreover, the subgroups with longer TTS included a significantly larger proportion of patients who had received more cycles of NCT and fewer cycles of ACT. These chemotherapy administration differences may have impact on the final oncological outcomes, it is noteworthy that the total number cycles of chemotherapy (combining NCT and ACT) received were similar among the 4 TTS subgroups. These variations were unavoidable given the retrospective nature of the present study. Finally, our study lacked data related to chemoradiotherapy and surgical complications, as well as to quality of life during and after treatment. This information may have been helpful in providing additional perspectives on different TTS and WPR.

## Conclusions

In patients with LARC, the initiation of neoadjuvant treatment and surgery of longer than 13 weeks is associated with preferable disease-free survival outcomes. The appropriate interval between the initiation of treatment and surgery, including the ideal waiting period between neoadjuvant radiotherapy and surgery, warrants further study.

## Data Availability Statement

All datasets generated for this study are included in the article/Supplementary Material.

## Ethics Statement

The study protocol was approved by the Central Ethics Committee of The Sixth Affiliated Hospital, Sun Yat-sen University (Guangzhou, China).

## Author Contributions

X-BW and FH designed the study. FH performed the contouring, treatment planning, and statistical analysis. QZ, JZ, YL, and PL reviewed the data. All authors discussed the data. FH and MC drafted the manuscript. All authors read and approved the final manuscript.

## Conflict of Interest

The authors declare that the research was conducted in the absence of any commercial or financial relationships that could be construed as a potential conflict of interest.

## Abbreviations

TME: total mesorectal excision
LARC: locally advanced rectal cancer
TTS: total-time-to-surgery
WPR: waiting-period-after-radiotherapy
TNT: total neoadjuvant therapy
NCT: neoadjuvant chemotherapy
ACT: adjuvant chemotherapy
DFS: disease-free survival
OS: overall survival
LRFS: local recurrence-free survival
DMFS: distant metastasis-free survival
pCR: pathologic complete response
AJCC: American Joint Commission on Cancer
CT: computed tomography
MRI: magnetic resonance imaging
EUS: endorectal ultrasonography
IMRT: intensity-modulated radiotherapy (IMRT)
GTV: gross tumor volume
CTV: clinical target volume
CRM: circumferential resection margin
HR: hazard ratio
CI: confidence interval
NCCN: National Comprehensive Cancer Network.
