# Supplementary material for: Prognostic Value of Interval Between the Initiation of Neoadjuvant Treatment to Surgery for Patients With Locally Advanced Rectal Cancer Following Neoadjuvant Chemotherapy, Radiotherapy and Definitive Surgery
Source: Front Oncol. 2020 Aug 21;10:1280. doi: 10.3389/fonc.2020.01280 (PMC7473386; doi:10.3389/fonc.2020.01280)
Supplement: Supplementary file 1 [file Table_1.DOCX]

**Supplementary Table 1.** Univariate and multivariate analysis of risk of death (overall survival [OS]), disease relapse (disease-free survival [DFS]), and distant metastasis (distant-metastasis-free survival [DMFS]) for 2267 patients with locally advanced rectal cancer, January 2010 through December 2018

| **Clinicopathological characteristics** | **Univariate analysis** | | | | | | | | | | |  | **Multivariate analysis** | | | | | | | | | | |
| --- | --- | --- | --- | --- | --- | --- | --- | --- | --- | --- | --- | --- | --- | --- | --- | --- | --- | --- | --- | --- | --- | --- | --- |
|  |  | 3-year OS |  |  |  | 3-year DFS |  |  | 3-year DMFS | | |  | 3-year OS | | |  |  | 3-year DFS |  |  |  | 3-year DMFS | |
|  | HR^a^ | 95%CI | *P* |  | HR^a^ | 95%CI | *P* |  | HR^a^ | 95%CI | *P* |  | HR^a^ | 95%CI | *P* |  | HR^a^ | 95%CI | *P* |  | HR^a^ | 95%CI | *P* |
| Age, >56 vs. ≤56 years | 1.181 | 0.939-1.485 | 1.181 |  | 0.971 | 0.810-1.165 | .752 |  | 1.121 | 0.928-1.354 | .235 |  | - | - | - |  | - | - | - |  | - | - | - |
| Gender, female vs. male | 1.104 | 0.861-1.416 | .436 |  | 1.096 | 0.905-1.328 | .348 |  | 0.962 | 0.787-1.176 | .708 |  | - | - | - |  | - | - | - |  | - | - | - |
| Clinical T stage |  |  | .148 |  |  |  | .211 |  |  |  | .228 |  |  |  |  |  |  |  |  |  |  |  |  |
| cT1 | 0.001 | 0.001-7.961 | .928 |  | 0.800 | 0.112-5.719 | .824 |  | 0.861 | 0.120-6.160 | .882 |  | - | - | - |  | - | - | - |  | - | - | - |
| cT2 | 0.321 | 0.118-0.870 | .025 |  | 0.641 | 0.347-1.183 | .155 |  | 0.711 | 0.385-1.316 | .278 |  | - | - | - |  | - | - | - |  | - | - | - |
| cT3 | 0.894 | 0.704-1.135 | .358 |  | 1.129 | 0.927-1.375 | .229 |  | 1.166 | 0.949-1.433 | .143 |  | - | - | - |  | - | - | - |  | - | - | - |
| cT4 | 1 | - | - |  | 1 | - | - |  | 1 | - | - |  | - | - | - |  | - | - | - |  | - | - | - |
| Clinical N stage |  |  | .420 |  |  |  | .030 |  |  |  | .030 |  |  |  |  |  |  |  | .023 |  |  |  | .021 |
| cN0 | 0.815 | 0.597-1.112 | .196 |  | 0.746 | 0.580-0.960 | .023 |  | 0.738 | 0.568-0.959 | .023 |  | - | - | - |  | 0.723 | 0.561-0.932 | .012 |  | 0.713 | 0.548-0.928 | .012 |
| cN1 | 0.902 | 0.697-1.167 | .433 |  | 0.803 | 0.656-0.983 | .034 |  | 0.796 | 0.646-0.981 | .033 |  | - | - | - |  | 0.811 | 0.662-0.993 | .043 |  | 0.801 | 0.649-0.988 | .038 |
| cN2 | 1 | - | - |  | 1 | - | - |  | 1 | - | - |  | - | - | - |  | 1 | - | - |  | 1 | - | - |
| Clinical TNM stage, II vs. III | 0.860 | 0.649-1.140 | .294 |  | 0.836 | 0.663-1.053 | .128 |  | 0.831 | 0.653-1.057 | .131 |  | - | - | - |  | - | - | - |  | - | - | - |
| Distal tumor distance from anal verge, *cm* |  |  | .348 |  |  |  | .032 |  |  |  | .039 |  |  |  |  |  |  |  | .095 |  |  |  | .054 |
| ≤5 | 1.033 | 0.529-2.017 | .924 |  | 1.130 | 0.634-2.013 | .679 |  | 1.140 | 0.624-2.084 | .670 |  | - | - | - |  | 1.081 | 0.606-1.926 | .793 |  | 1.097 | 0.600-2.005 | .764 |
| >5 and ≤10 | 0.826 | 0.416-1.637 | .583 |  | 0.849 | 0.472-1.529 | .586 |  | 0.867 | 0.469-1.602 | .648 |  | - | - | - |  | 0.819 | 0.454-1.476 | .506 |  | 0.841 | 0.455-1.556 | .582 |
| >10 | 0.821 | 0.306-2.206 | .696 |  | 1.283 | 0.607-2.713 | .514 |  | 1.415 | 0.656-3.049 | .376 |  | - | - | - |  | 1.194 | 0.563-2.529 | .644 |  | 1.328 | 0.615-2.868 | .470 |
| Unknown-Missing | 1 | - | - |  | 1 | - | - |  | 1 | - | - |  | - | - | - |  | 1 | - | - |  | 1 | - | - |
| Tumor differentiation |  |  | ＜.001 |  |  |  | .001 |  |  |  | .009 |  |  |  | ＜.001 |  |  |  | .001 |  |  |  | .008 |
| Well-differentiated | 0.778 | 0.532-1.138 | .196 |  | 0.788 | 0.532-1.138 | .140 |  | 0.755 | 0.539-1.057 | .101 |  | 0.799 | 0.545-1.173 | .252 |  | 0.793 | 0.578-1.090 | .153 |  | 0.757 | 0.541-1.061 | .106 |
| Moderately-differentiated | 0.568 | 0.432-0.747 | ＜.001 |  | 0.658 | 0.432-0.747 | ＜.001 |  | 0.692 | 0.547-0.875 | .002 |  | 0.560 | 0.426-0.736 | ＜.001 |  | 0.658 | 0.525-0.825 | ＜.001 |  | 0.690 | 0.545-0.873 | .002 |
| Poorly-differentiated | 1 | - | - |  | 1 | - | - |  | 1 | - | - |  | 1 | - | - |  | 1 | - | - |  | 1 | - | - |
| Neoadjuvant chemotherapy cycles, ≤3 vs. >3 | 1.557 | 1.195-2.029 | .001 |  | 1.151 | 0.950-1.396 | .151 |  | 1.135 | 0.931-1.385 | .211 |  | 0.924 | 0.738-1.158 | .494 |  | - | - | - |  | - | - | - |
| Total-time-to-surgery (TTS)^b^, *weeks* |  |  | .011 |  |  |  | ＜.001 |  |  |  | ＜.001 |  |  |  | ＜.001 |  |  |  | ＜.001 |  |  |  | ＜.001 |
| TTS-1, <13 | 1 | - | - |  | 1 | - | - |  | 1 | - | - |  | 1 | - | - |  | 1 | - | - |  | 1 | - | - |
| TTS-2, 13 to <15 | 0.812 | 0.612-1.077 | 0.149 |  | 0.791 | 0.631-0.992 | 0.042 |  | 0.752 | 0.594-0.951 | 0.018 |  | 0.775 | 0.613-0.980 | 0.033 |  | 0.798 | 0.636-1.002 | 0.052 |  | 0.759 | 0.599-0.962 | 0.022 |
| TTS-3, 15 to <17 | 0.585 | 0.410-0.834 | 0.003 |  | 0.546 | 0.413-0.723 | ＜0.001 |  | 0.552 | 0.415-0.734 | ＜0.001 |  | 0.520 | 0.383-0.706 | ＜0.001 |  | 0.547 | 0.413-0.724 | ＜0.001 |  | 0.552 | 0.415-0.735 | ＜0.001 |
| TTS-4, ≥17 | 0.675 | 0.480-0.949 | 0.024 |  | 0.701 | 0.542-0.906 | 0.007 |  | 0.632 | 0.483-0.828 | 0.001 |  | 0.658 | 0.493-0.880 | 0.005 |  | 0.680 | 0.525-0.880 | 0.003 |  | 0.613 | 0.468-0.803 | ＜0.001 |
| Waiting-period-after-radiotherapy (WPR)^c^, *weeks* |  |  | .452 |  |  |  | .312 |  |  |  | .279 |  |  |  |  |  |  |  |  |  |  |  |  |
| WPR-1, 4 to <6 | 0.818 | 0.460-1.456 | .495 |  | 1.036 | 0.640-1.678 | .885 |  | 1.237 | 0.736-2.080 | .423 |  | - | - | - |  | - | - | - |  | - | - | - |
| WPR-2, 6 to <8 | 0.672 | 0.423-1.066 | .092 |  | 0.831 | 0.572-1.207 | .332 |  | 1.027 | 0.682-1.548 | .897 |  | - | - | - |  | - | - | - |  | - | - | - |
| WPR-3, 8 to <10 | 0.673 | 0.418-1.083 | .103 |  | 0.887 | 0.607-1.297 | .536 |  | 1.085 | 0.716-1.644 | .702 |  | - | - | - |  | - | - | - |  | - | - | - |
| WPR-4, 10 to <12 | 0.734 | 0.404-1.335 | .311 |  | 0.650 | 0.396-1.065 | .087 |  | 0.714 | 0.417-1.224 | .220 |  | - | - | - |  | - | - | - |  | - | - | - |
| WPR-5, ≥12 | 1 | - | - |  | 1 | - | - |  | 1 | - | - |  | - | - | - |  | - | - | - |  | - | - | - |

Abbreviation: 95% CI, 95% Confidence interval.

^a^ Hazard ratios (HR) on 3-year overall survival (OS), disease-free survival (DFS), and distant-metastasis-free survival (DMFS) equate to relative risk of death, disease relapse, and distant metastasis, respectively.

^b^ Total-time-to-surgery (TTS) defined as time from initiation of neoadjuvant treatment to date of surgery.

^c^ Waiting-period-after-radiotherapy (WPR) defined as time from end of radiotherapy to date of surgery.
